# LC-MS-Based Untargeted Metabolomics Reveals Early Biomarkers in STZ-Induced Diabetic Rats With Cognitive Impairment

**DOI:** 10.3389/fendo.2021.665309

**Published:** 2021-06-30

**Authors:** Ruijuan Chen, Yi Zeng, Wenbiao Xiao, Le Zhang, Yi Shu

**Affiliations:** ^1^ Department of Geriatrics, Second Xiangya Hospital, Central South University, Changsha, China; ^2^ Department of Neurology, Xiangya Hospital, Central South University, Changsha, China; ^3^ Department of Neurology, Second Xiangya Hospital, Central South University, Changsha, China

**Keywords:** mild cognitive impairment (MCI), serum metabolomics, streptozotocin (STZ), biomarkers, diabetes mellitus (DM)

## Abstract

Diabetes in the elderly increases cognitive impairment, but the underlying mechanisms are still far from fully understood. A non-targeted metabolomics approach based on liquid chromatography-mass spectrometry (LC-MS) was performed to screen out the serum biomarkers of diabetic mild cognitive impairment (DMMCI) in rats. Total 48 SD rats were divided into three groups, Normal control (NC) group, high-fat diet (HFD) fed group and type 2 diabetes mellitus (T2DM) group. The T2DM rat model was induced by intraperitoneal administration of streptozotocin (STZ, 35 mg/kg) after 6 weeks of high-fat diet (HFD) feeding. Then each group was further divided into 4-week and 8-week subgroups, which were calculated from the time point of T2DM rat model establishment. The novel object recognition test (NORT) and the Morris water maze (MWM) method were used to evaluate the cognitive deficits in all groups. Compared to the NC-8w and HFD-8w groups, both NOR and MWM tests indicated significant cognitive dysfunction in the T2DM-8w group, which could be used as an animal model of DMMCI. Serum was ultimately collected from the inferior vena cava after laparotomy. Metabolic profiling analysis was conducted using ultra high performance liquid chromatography coupled with quadrupole time-of-flight mass spectrometry (UPLC-Q-TOF-MS) technology. Principal component analysis (PCA) and orthogonal partial least squares discriminant analysis (OPLS-DA) were used to verify the stability of the model. According to variable importance in the project (VIP > 1) and the p-value of t-test (P < 0.05) obtained by the OPLS-DA model, the metabolites with significant differences were screened out as potential biomarkers. In total, we identified 94 differentially expressed (44 up-regulated and 50 down-regulated) endogenous metabolites. The 10 top up-regulated and 10 top down-regulated potential biomarkers were screened according to the FDR significance. These biomarkers by pathway topology analysis were primarily involved in the metabolism of sphingolipid (SP) metabolism, tryptophan (Trp) metabolism, Glycerophospholipid (GP) metabolism, *etc.* Besides, SP metabolism, Trp metabolism and GP metabolism mainly belonging to the lipid metabolism showed marked perturbations over DMMCI and may contribute to the development of disease. Taken collectively, our results revealed that T2DM could cause cognitive impairment by affecting a variety of metabolic pathways especially lipid metabolism. Besides, serum PE, PC, L-Trp, and S1P may be used as the most critical biomarkers for the early diagnosis of DMMCI.

## Introduction

Increasing numbers of people are suffering from diabetes mellitus (DM), with the improvement of living standards and lifestyle changes. According to the eighth edition of the International Diabetes Federation (IDF), Diabetes Atlas in 2017, about 425 million people worldwide have diabetes, and the number is expected to rise to 700 million by 2045 (1). Besides, DM is considered to be a major disease associated with cognitive decline and dementia, another most common chronic disabling disease among the elderly, with a 1.5–2.5-fold higher risk of dementia than the general population (2, 3). So the high prevalence of diabetes-related cognitive dysfunction (DCD) will become a serious public health burden globally following significant financial and social implications. As dementia is an irreversible disease, early diagnosis and detection of dementia are critical for its prevention and treatment. However, there is still a lack of accurate and reliable diagnostic criteria for DCD, making early detection of diabetic cognitive impairment more difficult.

Growing studies have consistently proposed that Alzheimer’s disease (AD) is fundamentally a metabolic disease defined as “T3DM”, which has specific metabolic changes similar to the pathological characteristics of DM during the development of DCD (4). Recently, metabolomics as a powerful systematic approach born and defined in 1999 has been used frequently to evaluate global changes of disease-specific metabolites in biological samples (5). Compared with proteomics and genomics, metabonomics is characterized by high accuracy, high resolution, high sensitivity and small sample size, which is very helpful for discovering the pathophysiological changes of cells, body fluids, and tissues. As a result, it is an effective means of finding disease-related biomarkers that are more reliable and secure than genomics and proteomics (6). The most extensively applied techniques consist of nuclear magnetic resonance (NMR), gas chromatography (GC), and liquid chromatography-mass spectrometry (LC-MS) (7). In recent years, ultra high performance liquid chromatography coupled with quadrupole time-of-flight mass spectrometry (UPLC-Q-TOF-MS) has shown significant advantages in the accurate and rapid determination of metabolite activities (8).

In this paper, we used a combination of low-dose streptozotocin (STZ, 35 mg/kg body weight) and a high-fat diet (HFD, 60% of energy as fat) to establish a rat model mimicking human the T2DM model based on the previous study and observe its cognitive deficit (8). In this study, we aimed to primarily screen out the serum biomarkers for the early diagnosis of diabetic mild cognitive impairment (DMMCI) and explore its potential pathophysiological mechanism by analyzing the characteristics of the serum metabolomics in rats based on untargeted LC-MS technology. These differentially expressed metabolites could provide a novel strategy for the early diagnosis of DMMCI and give new insights into the pathophysiological changes and molecular mechanisms of disease in the future.

## Experiment

### Chemicals and Solutions (Chemicals and Reagents)

Streptozotocin (STZ, NO. S0130) was purchased from Sigma Corporation (St. Louis Missouri, USA). HFD (NO. D12492) containing 20% protein, 20% carbohydrate, and 60% fat was supplied by Research Diets, Inc. (New Brunswick, Canada). Blood glucose meter and test strip (GA-3, Sinocare Inc., China) were used to determine the random blood glucose (RBG) of tail venous blood in rats. Rat Insulin Elisa Kit (NO. 10-1250-01) was provided by Mercodia Inc. (Uppsala, Sweden). Acetonitrile, formic acid, methanol, and 2-Propanol using in High Pressure Liquid Chromatography (HPLC)-grade were purchased from Fisher Chemical (China).

### Animal Experiment

**Figure 1:**
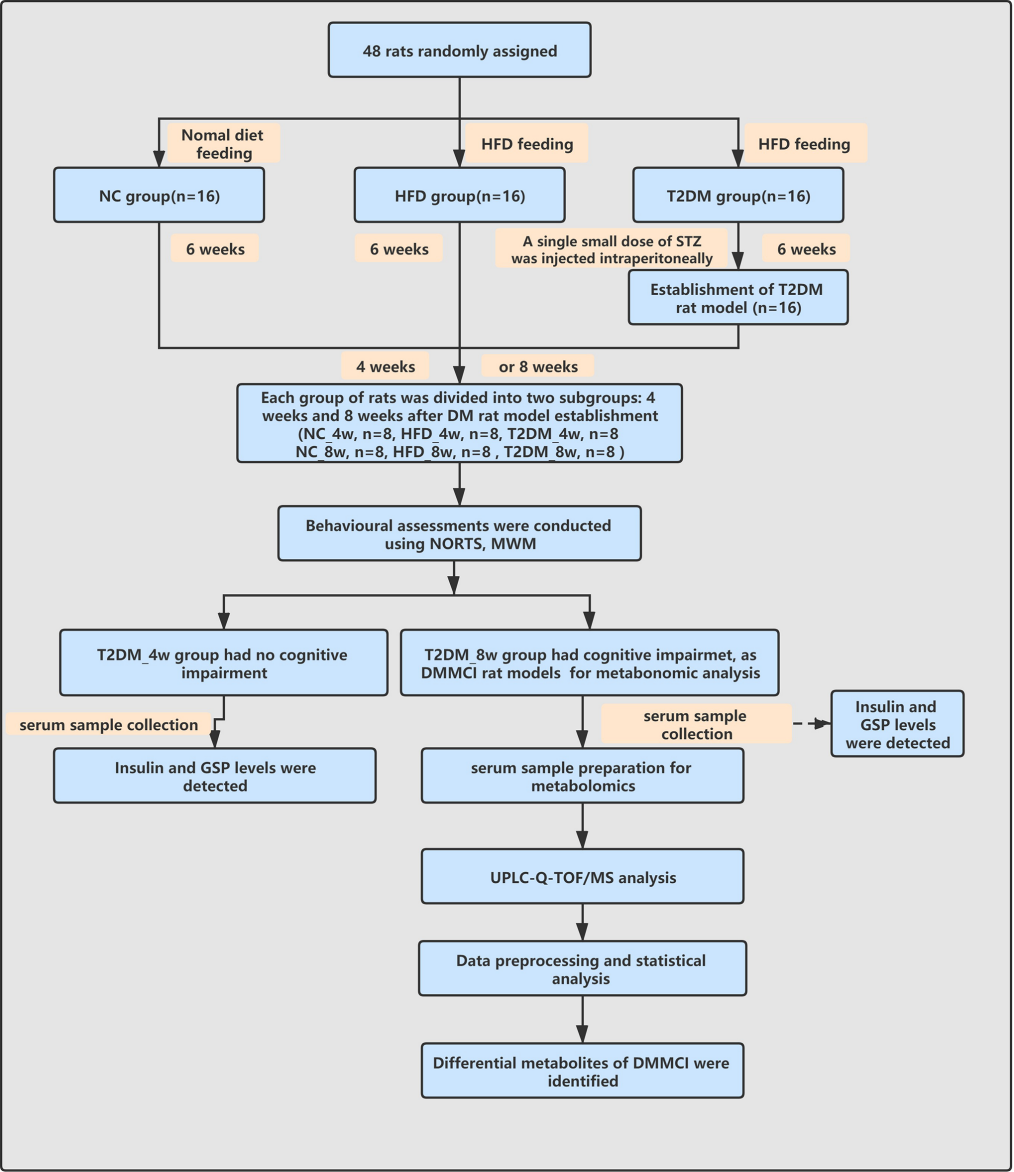
Work-flow diagram of the study process. NC, nomal control; HFD, high-fat diet; T2DM, type 2 diabetes mellitus; NORT, novel object recognition test MWM, Morris water maze; GSP, glycosylated serum protein; UPLC-Q-TOF-MS, ultra high performance liquid chromatography coupled with quadrupole time-of-flight mass spectrometry; DMMCI, diabetic mild cognitive impairment.

The work-flow of the study process was shown in **Figure 1**.

#### Animals, Diets, and Treatments

Total 48 healthy male Sprague–Dawley (SD) rats (aged 6–7 weeks) weighing 260 ± 20 g were purchased separately and reared in a specific pathogen-free (SPF) animal laboratory at the Experimental Animal Center of Central South University, China. All rats were maintained under controlled conditions (12 h light/dark cycles, 25°C, 50–60% room humidity) with food and water. To minimize the possible effects of circadian rhythm changes, all experiments were conducted at the same time in the morning. All research protocols were conducted according to the guide for the Care and Use of Laboratory Animals and approved by the Animal Ethics Committee of Central South University Xiangya School of Medicine.

#### HFD/STZ Induced T2DM Rat Model

For the experimental design, 48 rats were randomly divided into three equal groups as follows: Group I: normal control rats (NC, n = 16). Group II: HFD fed rats (HFD, n =16). Group III: T2DM rats (n = 16). The NC was fed a normal diet. The other group (HFD and T2DM) rats were fed with an HFD throughout the whole study containing 20% protein, 20% carbohydrate, and 60% fat (Research Diets, D12492, Canada) for 6 weeks. Then after 12 h of fasting, the rats of the T2DM group were injected with 35 mg/kg of streptozotocin (STZ; Sigma-Aldrich, USA) dissolved in a 0.1 M citric acid/sodium citrate buffer at pH 4.5 intraperitoneally (9). Two days after injection, rats with a constant RBG level ≥16.7 mmol/L were considered T2DM model rats and selected in further experiments. Then each group was further divided into 4-week (NC-4w, HFD-4w, and T2DM-4w) and 8-week (NC-8w, HFD-8w, and T2DM-8w) subgroups, which were calculated from the time point of T2DM rat model establishment. During each week, the body weights (BW) of the rats were measured until the end of the experiment, and the diabetes onset of the STZ injection group and RBG levels of all groups were determined using a blood glucose meter (GA-3 type Lifescan, Sinocare, China) with 2 μl blood collected from the tail veins.

#### Assessment of the HFD/STZ-Induced Diabetic Cognitive Impairment in the Diabetic Rat Model

All rats were assessed for cognitive behavioral deficits using the Morris water maze (MWM) test and novel object recognition test (NORT) at 11 or 15 weeks of the study (**Figure 1**).

##### NORT Task

The NORT relies on the rats’ innate tendency for investigating more novelty compared to a familiar object, which can be used to test rats’ non-spatial memory performances. As the procedure of NOR task previously reported (10), a rat was initially placed into an arena (50 cm long, 60 cm wide, 60 cm high) without objects for 2 min per day for 3 consecutive days. On the 4th day, each rat received two trials for a total duration of 15 minutes (min). The first trial (10 min) was the sample exploration which contained two identical objects placed in the left and right corners of the testing box. The second trial (5 min) was the testing trial when one of the two familiar objects presented during exploration was replaced with a new object after 1 h inter-trial interval. A video camera mounted above the testing box recorded the animal’s behavior once the rats were placed into the box for the object recognition test. The walls and floor of the testing box were cleaned with 70% isopropyl alcohol prior to each test, and the rats were returned to their home cages after each trial. Exploration behaviors were defined as a rat touching the object with its nose and/or directing towards the object within 2 cm. Each object exploration time was measured with a stopwatch and a discrimination ratio (DR) = [TN/(TF + TN), TF = time spent exploring familiar sample, TN = time spent exploring the novel object; DR was calculated to evaluate the recognition memory. A recognition DR significantly above 0.50 illustrates a novelty preference and positive recognition memory (11).

##### MWM Test

After the NOR test, the rats were subjected to 5 days of trial in the MWM tests to investigate their spatial learning ability and memory function after the object recognition test as described previously (12), which was developed by Richard Morris (13). In brief, the test was conducted in a round opaque pool (170 cm in diameter) filled with water (temperature 26 ± 1°C), virtually divided into four quadrants. The escape platform is a clear platform with a diameter of 10 cm, submerged about 1 cm beneath the surface of the water and located in the fixed target quadrant. The maze was surrounded by blue curtains, with visual stimuli of various shapes placed. Hidden platform test: each rat was trained for four consecutive days, four times one day by placing the animal into each quadrant as a starting point. Animals were given the 90 s per trial to locate the hidden platform, and any animal that did not find the platform within the 90 s was guided to the platform with sticks. Then they were set to remain on the platform for 15 s, regardless of where it was located. From the second day of training, behavioral parameters were recorded using an online image video tracking system (Stoelting Co., USA) within a maximum of 90 s as the escape latency in each trial. Spatial probe test: on the 5th day, the platform was removed from the pool. Each rat was left to the farthest quadrant of the pool from the primary platform. The probe time and the percentage of time spent in the target quadrant were tracked and analyzed by the tracking system.

### Sample Preparation and Determination of Hormonal and Biochemical Parameters

After 12 h of fasting, animals were anesthetized with chloral hydrate. The blood sample was immediately collected from the inferior vena cava after laparotomy. Following centrifugation at 3,000 g for 10 min, the serum was collected and stored at 80°C until use. Serum insulin levels were measured with the Rat Insulin Mercodia (Mercodia AB, Uppsala, Sweden) by enzyme-linked immunosorbent assay (ELISA) (Multiskan MK3, Thermo Scientific, USA). Glycated serum protein (GSP) was measured with a biochemical analyzer (Rayto Chemray 800, Shenzhen, China).

### Serum Sample Preparation for Metabolomics

A 100 ul liquid sample placed in a 1.5 ml centrifuge tube was added with 400 ul extract (acetonitrile: methanol = 1:1). The mixture was then injected with a 20 ul internal standard (IS, 0.3 mg/ml, containing L-2-chloro-phenylalanine and acetonitrile) and blended by vortex for 30 s and ultrasound (40 kHz, 5°C) for 30 min. The samples were settled at −20°C for 30 min to precipitate and obtained by centrifugation (13,000 g, 4°C) for 15 min; the supernatant was transferred, dried with nitrogen, and stored at −80°C for LC-MS/MS analysis.

Besides, as a necessary part of the quality control and system conditioning process, the quality control (QC) sample was made by mixing equal volumes of each sample. Resolution with 100 ul complex solution (acetonitrile: water = 1:1) was followed by low temperature ultrasonic extraction for 5 min (5°C, 40 k Hz). The mixture was centrifuged for 5 min (13,000 g, 4°C), and the supernatant was transferred to a sample injection vial with an inner cannula for analysis on the machine; 20 ul of the supernatant for each sample was transferred and mixed it as a QC sample. It was injected at regular intervals (every 9–10 samples) to minimize the carryover and monitor the stability of the experiment.

### UPLC-Q-TOF/MS Analysis

The metabolites were separated by chromatography on an ExionLTMAD system (AB Sciex, USA) which is equipped with an ACQUITY UPLC BEH C18 column (100 mm × 2.1 mm i.d., 1.7 µm; Waters, Milford, USA). The mobile phases contained two solvents [A: 0.1% formic acid in water with formic acid (0.1%), B: 0.1% formic acid in acetonitrile: isopropanol (1:1, v/v)]. The solvent gradient varies with the following conditions: a) 0–3 min, 95% (A): 5% (B) changed to 80% (A): 20% (B); b) 3–9 min, 80% (A): 20% (B) changed to 5% (A): 95% (B); c) 9–13 min, 5% (A): 95% (B) changed to 5% (A): 95% (B); d) 13–13.1 min, 5% (A): 95% (B) changed to 95% (A): 5% (B); e) 13.1–16 min, 95% (A): 5% (B) changed to 95% (A): 5% (B) for the systems equilibration. The injection volume of the sample was 20 ul with the flow rate at 0.4 ml/min, and the column temperature was set to hold at 40°C. All these samples were stored at 4°C during the period of analysis.

The positive and negative ion scanning modes were processed on the UPLC system to collect the quality spectrum signal of the sample, which was coupled to a quadrupole-time-of-flight mass spectrometer (Triple TOFTM5600+, AB Sciex, USA) equipped with an electrospray ionization (ESI) source. The detection was conducted over a mass range of 50–1,000 m/z. The optimal conditions included: ion-spray voltage floating (ISVF), 5,000 V in positive mode, −4000 V in negative mode; curtain gas (CUR), 30 psi; source temperature, 500°C; both ion source GS1 and GS2, 50 psi; declustering potential, 80 V; collision energy (CE), 20–60 V cyclic impact energy.

### Data Preprocessing and Annotation

Based on UPLC-Q-TOF/MS analysis, this paper imports the original data into Progenesis QI 2.3 (Nonlinear Dynamics, Waters, USA) for peak detection and calibration. The preprocessing results generated a data matrix, including retention time (RT), mass charge ratio (M/Z) values, and peak intensity. At least 80% of the metabolic features detected in any set of samples were retained. After screening, the minimum metabolic value was calculated for the specific samples whose metabolic level was lower than the quantitative lower limit, and the sum of all metabolic characteristics was normalized. The IS was used to evaluate the stability of the instrument. The pooled QC was not only used for the conditioning of the LC-MS system to ensure its stability before starting the analysis sequence, but also used as a powerful approach to tracking the intrabatch analytical variability with principal component analysis (PCA) plot visualization and setting standard deviation limits for selected features. The metabolic characteristics of QC greater than 30% relative standard deviation (RSD) are abandoned. After normalization and imputation, statistical analysis of log10-converted data was performed to determine significant differences in metabolite levels between the comparison groups. These metabolic characteristics were identified by precise mass spectrometry. Searching a reliable biochemical database such as the human metabolome database (HMDB) (http://www.hmdb.ca/) and Metlin database (https://metlin.scripps.edu/) MS/MS fragments’ spectra, accurate mass, and isotope ratio difference were obtained. For MS/MS confirmed metabolites, only metabolites with MS/MS fragment score greater than 50 are considered to be positively identified.

### Statistical Analysis

A multivariate statistical analysis including PCA and orthogonal least partial square discriminant analysis (OPLS-DA) was conducted using ropls (Version1.6.2, http://bioconductor.org/packages/release/bioc/html/ropls.html) R package. The stability of the model was assessed using seven cyclic interaction validations. Besides, a two-tailed student’s t-test combined with the multivariate analysis of OPLS-DA was conducted. The significantly different metabolites were selected based on the variable importance in the project (VIP) obtained by the OPLS-DA model and p-value of the student’s t-test. The metabolites with VIP >1 and p <0.05 (after Benjamini–Hochberg false discovery rate correction) were significantly different metabolites. Correlation analysis was performed using Pearson correlation test coefficient, and p-value <0.05 was considered significant between each comparison. Differential metabolites were mapped into the metabolic enrichment and pathway analysis through the KEGG database (https://www.kegg.jp/kegg/pathway.html). The Python package Scipy. stats (https://docs.scipy.org/doc/scipy/) performed a pathway enrichment analysis, and the biological pathway most relevant to the experimental treatment was identified using Fisher’s exact test. Significantly altered metabolite data were introduced for metabolic analysis 5.0 (https://www.metaboanalyst.ca) to investigate the DMMCI metabolic mechanisms.

Other statistical analyses were performed using Prism 5.0 (GraphPad) or the SPSS 11.0 software package. Data were expressed as the mean ± SEM. For the repeated-measures data such as weight, RBG levels, MWM data, a two-way repeated-measure (RM) ANOVA was performed. The remaining biochemical data such as insulin levels were analyzed by using the one-way ANOVA test or t-test. P <0.05 was considered statistically significant.

## Results

### The Establishment of the Animal Model for the Diabetic Cognitive Dysfunction

#### T2DM Rat Model Induced by HFD and STZ

Diabetic SD rat models induced by the administration of STZ in the 6th week were confirmed through monitoring BW, RBG levels, GSP levels, and insulin levels.

**Figure 2:**
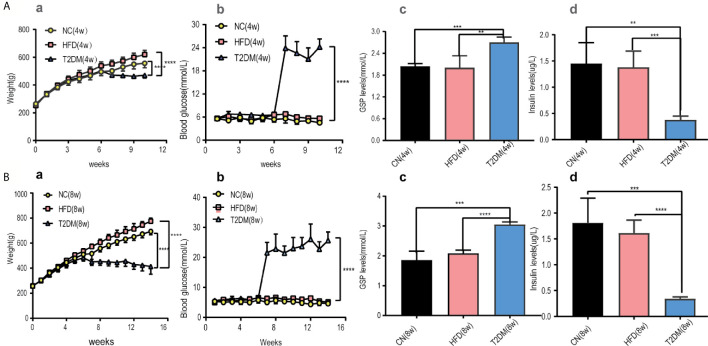
Diabetic rats was set up and treated with HFD and STZ. **(A)** Basic characteristics of rats in the 4-week subgroup, **(B)** Basic characteristics of rats in the 8-week subgroup. (a) Body weight, (b) Random blood glucose levels were measured at the indicated times each week in NC, HFD, and T2DM rats. (c) GSP levels, (d) insulin levels were determined 4 or 8 weeks after STZ injection. Mean ± S.E.M, n = 8. *p < 0.05; **p < 0.01;***p < 0.001;****p < 0.0001. NC, nomal control; HFD, high-fat; T2DM, type 2 diabetes mellitus; GSP, glycosylated serum protein.

The RBG and GSP levels of T2DM group (both in T2DM-4w and T2DM-8w) were significantly higher than those of the NC and HFD rats [RBG-4w: F (18, 150) = 104.1, P < 0.0001; RBG-8w: F (26, 210) = 56.82, P < 0.0001; GSP: T2DM-4w *vs* NC-4w, P < 0.001; T2DM-4w *vs* HFD-4w, P < 0.01; T2DM-8w *vs* NC-8w, P < 0.001; T2DM-8w *vs* HFD-8w, P < 0.0001]. The HFD fed rats gained more weight in the first 6 weeks than the NC group. After STZ administration, the body weight and insulin levels of the diabetic rats were significantly decreased compared to those of other groups [BW-4w: F (10, 110) = 10.14, P < 0.0001; BW-8w: F (14, 150) = 31.99, P < 0.0001; Insulin: T2DM-4w *vs* NC-4w, P < 0.01; T2DM-4w *vs* HFD-4w, P < 0.001; T2DM-8w *vs* NC-8w, P < 0.001; T2DM-8w *vs* HFD-8w, P < 0.0001]. Besides, the decrease of insulin levels was more obvious in the T2DM-8w group than in the T2DM-4w group. Persistent high glucose and GSP, as well as low insulin levels suggested the establishment of a diabetic rat model (**Figure 2**).

#### Results of DMMCI Assessments

**Figure 3:**
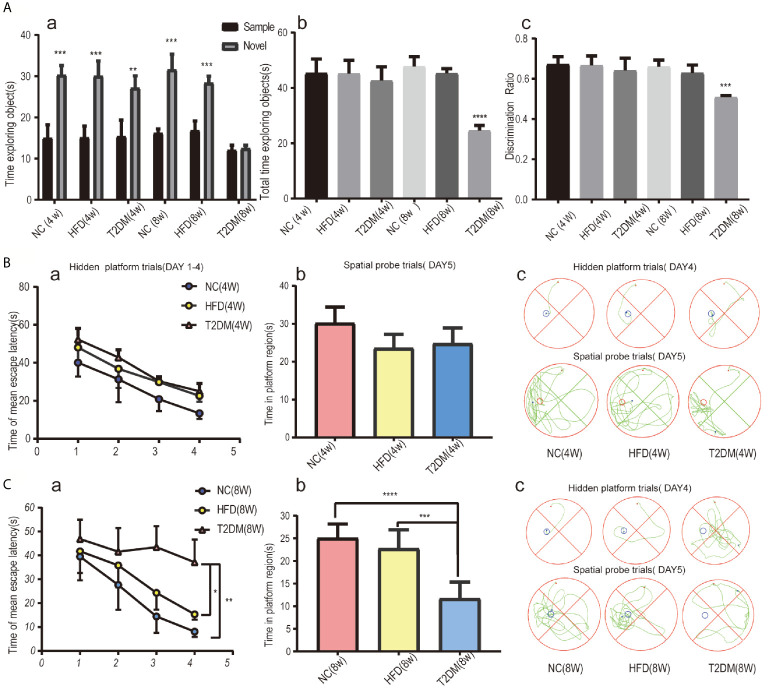
Assessments of mild cognitive impairment using NORT and MWM tests. Novel object recognition (NOR) test analysis revealed no evidence of deficits in short-term recognition memory in the T2DM-4w group of rats and significant impairment of cognitive function in the T2DM-8w group of rats; (a) TN(time spent exploring the novel object) vs TF(time spent exploring familiar sample) in each group, **p < 0.01; ***p < 0.001; (b) the average total exploration time(ATET) compared between T2DM-8w with NC-8w and HFD-8w group, ****p < 0.0001 (n = 4/group). **(A)** Spatial learning and memory evaluated by the MWM test in all 4w subgroups showed that T2DM-4w group had no cognitive impairment; **(B)** Spatial learning and memory evaluated by the MWM test in all 8w subgroups showed that T2DM-8w group had significant cognitive impairment; (**B,C**) (a) Mean escape latency during the hidden platform tests (DAYs 1–4); (b) The time in the target quadrant during the spatial probe tests(day5); (c) Representative searching strategy of rats on day 4 and day 5. *p < 0.05; **p < 0.01; ***p < 0.001;****p < 0.0001 (n = 4/group).

In the NOR tests (**Figure 3A**), the results of one-way analysis of variance showed that the average total exploration time (ATET) and DR of rats had no significant difference among the groups at 4w [ATET-4w, F (2, 9) = 0.3666, P > 0.05, DR-4w, F (2, 9) = 0.4388, P > 0.05], but had significant difference among the three groups at 8w [ATET-8w, F (2, 9) = 101.4, P < 0.0001, DR-8w, F (2, 9) = 26.93, P < 0.001]. The ATET and DR of the T2DM-8w group were significantly lower than those of the other two groups. Two-way ANOVA showed that, except for T2DM-8w rats, there were significant differences in the exploration time of familiar and novel things in other groups. Although T2DM-8w rats spent more time exploring novelty than familiarity, the difference was not significant (P > 0.05).

As shown in **Figure 3B**, the mean escape latency for the trained rats significantly decreased over the 4 days, and the total time spent in the target quadrant in the spatial probe trials without the platform on day 5 showed no significance in all 4w groups. It suggested no learning and memory deficits in T2DM-4w rats. However, in the 8w groups, the T2DM-8w rats performed significantly worse than the NC and HFD in the hidden platform trials (p < 0.0001, p < 0.01) and the probe trials (p < 0.01, P < 0.05) (**Figure 3C**). Though escape latency decreased significantly across the four days of training, there were significant differences between the three groups [F (3, 40) = 3.311, P < 0.05]. A Bonferroni *post-hoc* test revealed that the T2DM-8w group took longer to find the platform than the other two groups on both day 3 and day 4 (both, P < 0.0001). In the probe trials, we found a significant difference in the time spent in the target quadrant among the three groups [F (2, 15) = 23.03, P < 0.0001]. Turkey’s test indicated that the T2DM-8w group spent less time in this quadrant than the NC and HFD groups (P < 0.0001, P < 0.001), but there was no difference between the NC and HFD groups in swimming time (P > 0.05).

Both NOR and MWM tests indicated significant cognitive dysfunction in the T2DM-8w group, which could be used as an animal model of DMMCI for subsequent metabolomic studies to search for potential metabolomic markers.

### Serum Metabolic Profiling by UHPLC-Q-TOF/MS in DMMCI Rats

#### Identification of Potential Biomarkers of DMMCI

**Figure 4:**
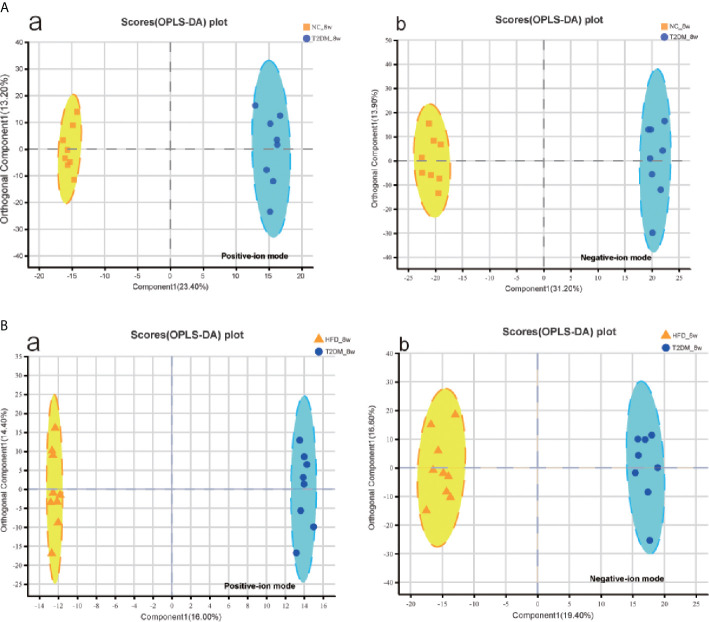
Multivariate statistical analysis of serum metabolomics of the 8w subgroups of rats after the establishment of T2DM rat model. **(A, B)** OPLS-DA score plots between every two groups(T2DM *vs* NC, T2DM *vs* HFD) in positive- and negative-ion modes, respectively.

**Figure 5:**
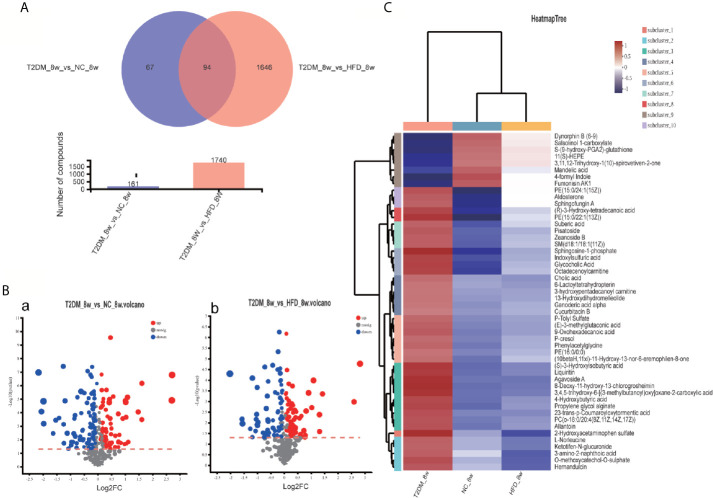
Venn diagram, volcanic plot and heat map of the identified differentially expressed metabolites. **(A)** Venn diagram of 94 differentially expressed metabolites through the comparison between T2DM with groups NC and HFD; **(B)** Volcano plot of differentially expressed metabolites in T2DM group compared with NC and HFD groups, respectively. Volcano plots were constructed using fold‐change values and p‐values. The vertical lines correspond to 2.0 fold-up and down-regulation between each group (T2DM *vs*. NC, T2DM *vs* HFD), and the horizontal lines represent p‐values. Red plot points represent up-regulated metabolites with statistical significance. Blue plot points represent down-regulated metabolites with statistical significance. Gray plot points represent no significant metabolites; **(C)** Heat map analysis of 94 differential metabolites identified between T2DM, NC, and HFD groups. The blue band indicates a decreased level of metabolite, and the red band indicates an increased level of metabolite.

The UPLC-Q-TOF/MS in metabolomics was applied to detect and collect the metabolic profiles of serum samples in positive and negative ion modes between the three groups. PCA method was used to find abnormal samples and evaluate the repeatability of experimental data. PCA score chart results show a high degree of QC polymerization, indicating good QC repeatability and a stable analysis system (**Figure S1**). Multivariate statistical analysis by OPLS-DA supervised pattern recognition method was adopted to identify the metabolomic differences of serum in three rat groups. As shown in **Figure 4**, significantly separated clusters appeared between every two groups (DMMCI *vs* NC, DMMCI *vs* HFD) in both positive ion and negative ion modes, respectively, which indicated that the serum metabolic profiles were different at baseline. High statistical values of R2Y and Q2 in the OPLS-DA score plots indicated the fitness and the prediction ability of our model [DMMCI *vs* NC, positive-ion, R2 = (0, 0.8406), Q2 = (0, −0.5128), negative-ion, R2 = (0, 0.8081), Q2 = (0, −0.3076); DMMCI *vs* HFD, positive-ion, R2 = (0, 0.9882), Q2 = (0, −0.2121), negative-ion, R2 = (0, 0.9595), Q2 = (0, −0.1083)]. Subsequently, potential markers of DMMCI (DM-8w) were screened for further study based on the ions with VIPs >1.0 and P <0.05 after OPLS-DA analysis by comparing them with those of NC and HFD groups, respectively (shown in **Figure 5A**). A total of 94 differentially expressed (44 up-regulated and 50 down-regulated) endogenous metabolites were discovered as shown in **Figure 5A**, **Tables 1**, **2**. In the positive ion mode, 43 (24 up-regulated/19 down-regulated) differential endogenous metabolites were putatively identified. In the negative ion mode, 51 (20 up-regulated/31 down-regulated) differential metabolites were detected. Hierarchical cluster analysis was used to further characterize the specific and unique expression patterns of these differentially expressed metabolites in serum of NC, HFD, and DMMCI rats (**Figure 5B**), showing a global profile of all serum metabolites that have been detected and visualized. Cluster heat map analysis of 94 differential metabolites showed clear separation for each alignment. Interestingly, differences in metabolite heat maps between groups of rats based on DMMCI and NC/HFD showed clear clustering. This study indicated the reliability of the OPLS-DA model for distinguishing different disease-specific metabolic phenotypes (**Figure 5C**). The metabolites with similar variation trends in abundance were located closer, indicating that the metabolites of DMMCI were clustered closely and separated from other groups. The 44 up-regulated and 50 down-regulated differential metabolites were ranked according to the FDR (corrected P-value) of significance and the top 10 significant metabolites were selected as the potential biomarkers, separately. *Up-regulated* markers included PE [15:0/22:1 (13Z)], 3-[8-hydroxy-2-methyl-2-(4-methylpent-3-en-1-yl)-2H-chromen-5-yl]propanoic acid, Sphingosine-1-phosphate (S1P), PE [15:0/24:1(15Z)], 3,4,5-trihydroxy-6-[(3-methylbutanoyl)oxy]oxane-2-carboxylic acid, Agavoside A, 2-Hydroxyacetaminophen sulfate, Propylene glycol alginate, Glycocholic Acid, Sphingofungin A. *Down-regulated* markers included PC [14:0/22:5(4Z,7Z,10Z,13Z,16Z)], LysoPC [20:5(5Z,8Z,11Z,14Z,17Z)], N-Arachidonoyl-L-Serine, Sagittariol, (±)12-HEPE, LysoPE [0:0/20:1(11Z)], (±)12,13-DiHOME, LysoPC [18:3(9Z,12Z,15Z)], LysoPC [16:1(9Z)/0:0], LysoPE [0:0/20:2(11Z,14Z)].

**Table 1:** List of differentially expressed (*up-regulated*) endogenous metabolites detected by UHPLC-QTOF/MS in the T2DM(8W) group compared with NC(8w) and HFD(8w) groups.

	Metabolite	Library ID	Mode	NC(8w) mean ± SD	HFD(8w) mean ± SD	T2DM(8w) mean ± SD	P-value	FDR
1	PE [15:0/22:1(13Z)]	HMDB0008908	neg	4.148 ± 0.09274	4.326 ± 0.128	4.564 ± 0.0801	6.164E-07	0.00002617
2	3-[8-hydroxy-2-methyl-2-(4-methylpent-3-en-1-yl)-2H-chromen-5-yl]propanoic acid	HMDB0134704	neg	0.6685 ± 0.4295	1.634 ± 0.4523	2.042 ± 0.1598	0.000008904	0.0002114
3	Sphingosine-1-phosphate	LMSP01050001	pos	4.164 ± 0.1283	4.284 ± 0.1252	4.607 ± 0.1008	0.000004242	0.0006402
4	PE [15:0/24:1(15Z)]	HMDB0008915	neg	3.209 ± 0.1809	3.453 ± 0.1975	3.694 ± 0.09474	0.00004521	0.0006738
5	3,4,5-trihydroxy-6-[(3-methylbutanoyl)oxy]oxane-2-carboxylic acid	HMDB0130798	pos	2.026 ± 0.1621	2.04 ± 0.2671	2.525 ± 0.086	0.00000554	0.000785
6	Agavoside A	HMDB0034391;LMST01080006	pos	0.8463 ± 0.3096	0.8432 ± 0.4648	1.796 ± 0.2367	0.00001012	0.001119
7	2-Hydroxyacetaminophen sulfate	HMDB0062547	neg	0.6808 ± 0.4383	0.2935 ± 0.4622	2.075 ± 0.7116	0.0001569	0.001652
8	Propylene glycol alginate	HMDB0039860	pos	1 ± 0.3304	1.141 ± 0.633	2.009 ± 0.288	0.00004428	0.002477
9	Glycocholic acid	HMDB0000138;HMDB0000331;LMST05030001	pos	3.361 ± 0.4004	3.691 ± 0.508	4.492 ± 0.306	0.00005172	0.002637
10	Sphingofungin A	LMSP01080061	pos	0.3903 ± 0.7135	1.488 ± 1.261	2.54 ± 0.5903	0.00006245	0.002737
11	Suberic acid	LMFA01170001;HMDB0000893	neg	2.522 ± 0.149	2.73 ± 0.414	3.091 ± 0.2445	0.0003707	0.003135
12	Aldosterone	HMDB0000037;LMST02030026	pos	1.298 ± 0.4807	1.96 ± 0.7199	2.634 ± 0.3457	0.00008335	0.003312
13	(R)-3-Hydroxy-tetradecanoic acid	HMDB0010731	neg	1.878 ± 0.2754	2.35 ± 0.3804	3.152 ± 0.6299	0.0004312	0.003472
14	Liquiritin	LMPK12140021;HMDB0029520	pos	4.194 ± 0.1063	4.216 ± 0.1664	4.512 ± 0.1121	0.000107	0.003839
15	Pisatoside	HMDB0039127	pos	2.157 ± 0.09419	2.223 ± 0.2149	2.447 ± 0.09779	0.0001321	0.004438
16	(S)-3-Hydroxyisobutyric acid	HMDB0000023	pos	2.363 ± 0.06227	2.388 ± 0.1694	2.694 ± 0.1328	0.0001327	0.004438
17	8-Deoxy-11-hydroxy-13-chlorogrosheimin	HMDB0041037	pos	2.776 ± 0.08043	2.786 ± 0.1374	3.131 ± 0.1458	0.0001349	0.004444
18	Octadecenoylcarnitine	HMDB0094687	pos	4.24 ± 0.0814	4.374 ± 0.176	4.664 ± 0.186	0.0002363	0.006075
19	SM [d18:1/18:1(11Z)]	HMDB0012100	neg	1.805 ± 0.5173	2.021 ± 0.4085	2.547 ± 0.1286	0.001258	0.008047
20	Zeanoside B	HMDB0038844	neg	1.688 ± 0.3638	1.865 ± 0.4262	2.338 ± 0.215	0.001518	0.009299
21	O-methoxycatechol-O-sulphate	HMDB0060013	neg	2.081 ± 0.7052	1.731 ± 0.6763	3.247 ± 0.7041	0.001631	0.009696
22	Blepharin	HMDB0029344	neg	1.313 ± 0.5386	2.06 ± 0.2259	2.469 ± 0.4154	0.001885	0.01074
23	Indoxylsulfuric acid	HMDB0000682	neg	4.131 ± 0.2206	4.268 ± 0.1585	4.684 ± 0.2737	0.002276	0.01251
24	4-Hydroxybutyric acid	HMDB0000710	pos	1.933 ± 0.2757	2.009 ± 0.3993	2.641 ± 0.3109	0.0007537	0.01308
25	3-amino-2-naphthoic acid	–	neg	1.008 ± 0.6616	0.6332 ± 0.4615	1.701 ± 0.5239	0.002568	0.01366
26	PC [o-18:0/20:4(8Z,11Z,14Z,17Z)]	LMGP01020247;HMDB0013420	neg	2.846 ± 0.1666	2.872 ± 0.1676	3.145 ± 0.1503	0.003204	0.01611
27	Allantoin	HMDB0000462	neg	2.451 ± 0.1714	2.487 ± 0.1535	2.757 ± 0.1545	0.003315	0.01651
28	Hernandulcin	HMDB0037906	neg	1.876 ± 0.1966	1.754 ± 0.2666	2.207 ± 0.2097	0.003415	0.0169
29	23-trans-p-Coumaroyloxytormentic acid	HMDB0040682	pos	2.174 ± 0.2028	2.18 ± 0.2143	2.569 ± 0.2203	0.003532	0.03302
30	L-Norleucine	HMDB0001645	pos	3.127 ± 0.2053	3.051 ± 0.3139	3.458 ± 0.1724	0.003686	0.03413
31	(E)-3-methylglutaconic acid	LMFA01170068	pos	1.697 ± 0.1063	1.718 ± 0.1751	1.92 ± 0.1195	0.004254	0.03736
32	Cholic acid	LMST04010001;HMDB0000619	neg	4.675 ± 0.4112	4.64 ± 0.5403	5.249 ± 0.3615	0.01501	0.04857
33	Ketotifen-N-glucuronide	HMDB0060596	pos	1.708 ± 0.223	1.621 ± 0.355	2.088 ± 0.2461	0.008069	0.05475
34	9-Oxohexadecanoic acid	HMDB0030973	neg	2.275 ± 0.1388	2.376 ± 0.5223	3.179 ± 0.7307	0.02066	0.06055
35	PE (16:0/0:0)	LMGP02050002;HMDB0011503	pos	4.191 ± 0.2451	4.242 ± 0.2388	4.472 ± 0.09897	0.01097	0.06678
36	Ganoderic acid alpha	HMDB0033024	pos	2.223 ± 0.8058	2.313 ± 0.6968	3.007 ± 0.1928	0.0116	0.06964
37	P-Tolyl sulfate	–	neg	3.999 ± 0.6002	4.066 ± 0.4533	4.817 ± 0.6236	0.03111	0.07997
38	P-cresol	HMDB0001858	neg	2.463 ± 0.7798	2.619 ± 0.6048	3.514 ± 0.768	0.03305	0.08354
39	(10betaH,11xi)-11-Hydroxy-13-nor-6-eremophilen-8-one	HMDB0037605	neg	2.586 ± 0.2696	2.71 ± 0.2906	3.049 ± 0.348	0.03411	0.08534
40	3-hydroxypentadecanoyl carnitine	HMDB0061641	pos	1.504 ± 0.9274	1.526 ± 1.032	2.536 ± 0.4998	0.01631	0.08538
41	6-Lactoyltetrahydropterin	HMDB0002065	pos	2.081 ± 0.28	2.062 ± 0.3702	2.437 ± 0.247	0.02741	0.1156
42	Phenylacetylglycine	HMDB0000821	pos	3.015 ± 0.4174	3.142 ± 0.3587	3.717 ± 0.5212	0.0045	0.122
43	Cucurbitacin B	HMDB0034927;LMST01010104	pos	1.761 ± 0.7552	1.828 ± 0.515	2.418 ± 0.4032	0.03453	0.1356
44	13-Hydroxydihydromelleolide	HMDB0036929	pos	2.415 ± 0.6791	2.454 ± 0.7022	3.241 ± 0.6334	0.04166	0.1539

The “-” indicates that the corresponding metabolite did not pass through the screening process. FDR represents the P-value corrected. Mean represents the average relative abundance of metabolites in different groups; SD represents standard deviation; one-way ANOVA was used to compare the three groups. P-value <0.05 is significant.

**Table 2:** List of differentially expressed (*down-regulated*) endogenous metabolites detected by UHPLC-QTOF/MS in the T2DM-8w group compared with NC-8w, and HFD-8w groups.

	Metabolite	Library ID	Mode	NC-8w mean ± SD	HFD-8w mean ± SD	T2DM-8w mean ± SD	P-value	FDR
1	PC [14:0/22:5(4Z,7Z,10Z,13Z,16Z)	LMGP01012130;HMDB0007890	neg	4.148 ± 0.1066	4.086 ± 0.1375	3.597 ± 0.09977	6.05E-08	0.000004795
2	LysoPC [20:5(5Z,8Z,11Z,14Z,17Z)]	HMDB0010397	neg	4.499 ± 0.1749	4.381 ± 0.2234	3.743 ± 0.1392	2.62E-07	0.00001466
3	N-Arachidonoyl-L-Serine	–	neg	3.118 ± 0.1499	2.855 ± 0.1767	2.409 ± 0.1316	2.80E-07	0.00001527
4	Sagittariol	HMDB0036835	neg	2.039 ± 0.284	1.708 ± 0.4858	0.8501 ± 0.1322	3.79E-07	0.00001851
6	(±)12-HEPE	–	neg	3.901 ± 0.1387	3.705 ± 0.1464	3.341 ± 0.09963	0.000000608	0.00002608
7	LysoPE [0:0/20:1(11Z)]	LMGP02050046;HMDB0011482	neg	3.558 ± 0.1086	3.44 ± 0.1151	3.233 ± 0.05414	0.000007621	0.0001873
5	(±)12,13-DiHOME	–	pos	2.232 ± 0.3281	2.024 ± 0.6836	0.4983 ± 0.3734	4.11E-07	0.0002344
8	LysoPC [18:3(9Z,12Z,15Z)]	HMDB0010388	neg	4.304 ± 0.09767	4.244 ± 0.1491	3.891 ± 0.1256	0.00001294	0.0002819
9	LysoPC [16:1(9Z)/0:0]	HMDB0010383	neg	4.919 ± 0.1613	4.749 ± 0.2127	4.458 ± 0.06338	0.00001682	0.0003518
10	LysoPE [0:0/20:2(11Z,14Z)]	LMGP02050047;HMDB0011483	neg	2.797 ± 0.1183	2.67 ± 0.1648	1.986 ± 0.2883	0.00002515	0.0004517
11	PC [16:0/18:3(6Z,9Z,12Z)]	LMGP01010598;HMDB0007974	neg	3.909 ± 0.1288	3.821 ± 0.1233	3.544 ± 0.1034	0.00004042	0.0006245
12	16-Hydroxy-10-oxohexadecanoic acid	HMDB0041287	neg	1.872 ± 0.1299	1.842 ± 0.2167	1.117 ± 0.2812	0.00004262	0.000649
13	LysoPC [20:1(11Z)]	HMDB0010391	neg	3.336 ± 0.1033	3.251 ± 0.08911	3.061 ± 0.08274	0.0000953	0.001158
14	LysoPC[20:2(11Z,14Z)]	HMDB0010392	neg	4.331 ± 0.2004	4.212 ± 0.2266	3.843 ± 0.1405	0.0001242	0.001381
15	Dynorphin B (6-9)	HMDB0012937	neg	2.649 ± 0.1651	1.995 ± 0.793	1.157 ± 0.6269	0.0001412	0.001521
16	LysoPC [20:3(5Z,8Z,11Z)]	HMDB0010393;LMGP01050139	neg	4.73 ± 0.1409	4.753 ± 0.1737	4.31 ± 0.1777	0.0001842	0.001853
17	Fumonisin AK1	HMDB0033397	neg	3.418 ± 0.1705	3.201 ± 0.2073	2.975 ± 0.1387	0.000234	0.00221
18	PC [17:1(9Z)/0:0]	LMGP01050126	pos	3.962 ± 0.1284	3.845 ± 0.1359	3.642 ± 0.03797	0.00003614	0.00226
19	Phytolaccinic acid	HMDB0034640	pos	4.158 ± 0.2234	4.029 ± 0.2471	3.626 ± 0.1074	0.00005294	0.002637
20	4-formyl Indole	–	pos	2.016 ± 0.09908	1.779 ± 0.178	1.537 ± 0.1904	0.00007242	0.003018
21	Prostaglandin F2a	HMDB0001139;LMFA03010002	neg	1.84 ± 0.06677	1.619 ± 0.2139	1.211 ± 0.3284	0.0004464	0.003581
22	1-(8Z,11Z,14Z-eicosatrienoyl)-sn-glycero-3-phosphocholine	–	pos	4.889 ± 0.209	4.949 ± 0.1933	4.466 ± 0.1559	0.00009844	0.003692
23	({6-[(E)-2-methoxyethenyl]-2-oxo-2H-chromen-7-yl}methoxy)sulfonic acid	HMDB0135800	neg	1.634 ± 0.1493	2.054 ± 0.4748	0.9036 ± 0.403	0.0004906	0.003854
24	2-O-beta-D-Glucopyranuronosyl-D-mannose	HMDB0039722	pos	2.707 ± 0.255	2.012 ± 1.022	0.6856 ± 0.8457	0.0001568	0.004829
25	Undecylenic acid	HMDB0033724;LMFA01030036	neg	1.398 ± 0.4536	1.491 ± 0.1991	0.9852 ± 0.2044	0.0007431	0.005375
26	1-Heptadecene-4,6-diyne-3,9-diol	LMFA05000599;HMDB0038782	neg	1.489 ± 0.4433	1.139 ± 0.4965	0.5592 ± 0.3104	0.0007936	0.005653
27	S-(9-hydroxy-PGA2)-glutathione	HMDB0013060	neg	2.483 ± 0.1517	2.07 ± 0.5766	1.396 ± 0.583	0.0009696	0.006584
28	N-(1-Deoxy-b-D-fructopyranosyl) (R)C(S)S-alliin	HMDB0040829	pos	1.63 ± 0.6413	1.161 ± 0.6927	0.3917 ± 0.02535	0.0003851	0.008527
29	5-Tetradecenoic acid	HMDB0000499	neg	1.782 ± 0.1411	1.734 ± 0.09607	1.464 ± 0.1496	0.001364	0.00858
30	Salsolinol 1-carboxylate	HMDB0013068	neg	2.107 ± 0.4538	1.524 ± 0.6139	0.7277 ± 0.7035	0.001511	0.009284
31	Artabsin	HMDB0036641	neg	2.099 ± 0.1712	2.026 ± 0.2375	1.763 ± 0.1536	0.002986	0.01525
32	LysoPE[0:0/18:1(11Z)]	HMDB0011475;LMGP02050039	neg	2.77 ± 0.2145	2.693 ± 0.2234	2.277 ± 0.2685	0.003575	0.01741
33	3,4,5-trihydroxy-6-[2-(3-oxoprop-1-en-1-yl)phenoxy]oxane-2-carboxylic acid	HMDB0134044	neg	2.123 ± 0.295	2.3 ± 0.2116	1.484 ± 0.5169	0.004692	0.02112
34	L-tryptophan	HMDB0000929	pos	4.831 ± 0.0709	4.782 ± 0.1133	4.6 ± 0.1235	0.001951	0.0227
35	Mandelic acid	HMDB0000703	pos	1.664 ± 0.7677	0.9643 ± 0.6709	0.4234 ± 0.2473	0.002352	0.02551
36	N-(1-Deoxy-1-fructosyl)threonine	HMDB0037843	neg	1.653 ± 0.7827	1.283 ± 0.6896	0.5343 ± 0.4483	0.006621	0.02742
37	6-Hydroxy-1H-indole-3-acetamide	HMDB0031173	neg	1.872 ± 0.7703	1.948 ± 0.6647	0.9572 ± 0.5212	0.007763	0.03038
38	LysoPE(0:0/20:0)	HMDB0011481;LMGP02050045	pos	3.014 ± 0.2419	2.846 ± 0.1636	2.567 ± 0.2013	0.004034	0.0361
39	Methyl N-methylanthranilate	HMDB0034169	pos	2.226 ± 0.1137	2.148 ± 0.2091	1.951 ± 0.1518	0.004539	0.0393
40	Baicalin	HMDB0041832;LMPK12111081	neg	2.626 ± 0.227	2.86 ± 0.368	1.802 ± 0.7688	0.01399	0.04611
41	3,11,12-Trihydroxy-1(10)-spirovetiven-2-one	HMDB0038154	pos	1.94 ± 0.2031	1.707 ± 0.2802	1.315 ± 0.4251	0.007232	0.05121
42	Isoquinoline	HMDB0034244	pos	1.952 ± 0.549	1.795 ± 0.6743	0.9008 ± 0.6406	0.009656	0.06099
43	Nopalinic acid	HMDB0029437	neg	2.7 ± 0.3405	2.642 ± 0.3953	1.732 ± 0.7764	0.02197	0.06305
44	Acetylcholine	HMDB0000895	pos	2.43 ± 0.1702	2.461 ± 0.1738	2.104 ± 0.2401	0.01098	0.06678
45	5-Methoxyindoleacetate	HMDB0004096	pos	1.982 ± 0.4658	1.658 ± 0.3664	1.08 ± 0.5623	0.01453	0.08067
46	Ganglioside GT3 (d18:1/20:0)	HMDB0012073	neg	2.608 ± 0.2643	2.482 ± 0.3186	2.066 ± 0.4347	0.03383	0.08495
47	11(S)-HEPE	–	pos	3.407 ± 0.1163	3.323 ± 0.0596	3.194 ± 0.1272	0.01611	0.08509
48	Nicotyrine	–	pos	1.521 ± 0.7153	1.522 ± 0.5714	0.7747 ± 0.451	0.01724	0.08768
49	Arginyl-Cysteine	HMDB0028706	pos	2.391 ± 0.4847	2.37 ± 0.4952	1.567 ± 0.5999	0.01841	0.09109
50	Phe4Cl-Phe-OH	–	pos	1.916 ± 0.1608	1.81 ± 0.6848	1.084 ± 0.7053	0.02812	0.118

The “-” indicates that the corresponding metabolite did not pass through the screening process. FDR represents the P-value corrected. Mean represents the average relative abundance of metabolites in different groups; SD represents standard deviation; one-way ANOVA was used to compare the three groups. P-value < 0.05 is significant.

#### KEGG Pathway Enrichment Analysis

**Figure 6:**
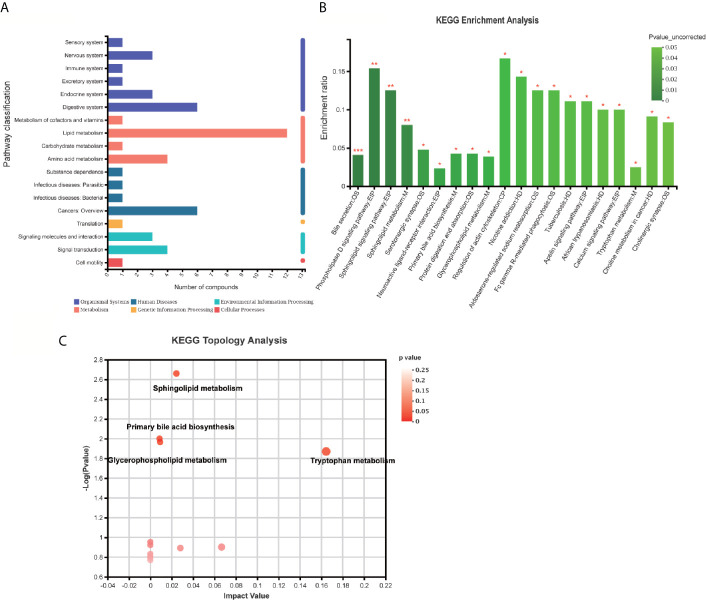
KEGG enrichment analysis and pathway analysis of the identified 94 differentially expressed metabolites. **(A)** Pathway classification analysis by KEGG revealed showed 12 metabolites annotated lipid metabolism, six metabolites annotated cancers: Overview, six metabolites annotated digestive system, four metabolites annotated signal transduction, four metabolites annotated amino acid metabolism, three metabolites annotated signaling molecules and interaction, three metabolites annotated endocrine system, three metabolites annotated nervous system, *etc.* **(B)** Pathway annotation analysis by KEGG revealed the pathways where p-value is in the top 20 including bile secretion: OS, Phospholipase D signaling pathway: EIP, sphingolipid signaling pathway: EIP, sphingolipid metabolism: M, Serotonergic synapse: OS, neuroactive ligand–receptor interaction: EIP, primary bile acid biosynthesis: M, protein digestion and absorption: OS, Glycerophospholipid (GP) metabolism: M, regulation of actin cytoskeleton: CP, nicotine addiction: HD, Aldosterone-regulated sodium reabsorption: OS, Fc gamma R-mediated phagocytosis: OS, tuberculosis: HD, Apelin signaling pathway: EIP, African trypanosomiasis: HD, calcium signaling pathway: EIP, tryptophan metabolism: M, choline metabolism in cancer: HD, cholinergic synapse: OS. **(B, C)** Pathway topology analysis highlighted the following pathways: sphingolipid (SP) metabolism, tryptophan (Trp) metabolism, Glycerophospholipid (GP) metabolism, primary bile acid biosynthesis, folate biosynthesis, Aminoacyl-tRNA biosynthesis, Valine, leucine, and isoleucine degradation, phenylalanine metabolism, glycine, serine, and threonine metabolism, Butanoate metabolism, arachidonic acid metabolism, steroid hormone biosynthesis, phenylalanine, tyrosine, and Trp biosynthesis, etc. *p < 0.05; **p < 0.01; ***p < 0.001.

Pathway classification analysis of the 94 differential metabolites by KEGG showed 12 metabolites annotated lipid metabolism (including Glycocholic Acid, S1P, Aldosterone, L-tryptophan, Acetylcholine, Cholic acid, LysoPC [20:5(5Z,8Z,11Z,14Z,17Z)], LysoPC [20:3(5Z,8Z,11Z)], LysoPC [20:2(11Z,14Z)], SM [d18:1/18:1(11Z)], LysoPC [20:1(11Z)], LysoPC [16:1(9Z)/0:0, Prostaglandin F2a], six metabolites annotated cancers: overview (L-tryptophan), LysoPC [20:5(5Z,8Z,11Z,14Z,17Z)], LysoPC [20:3(5Z,8Z,11Z)], LysoPC [20:2(11Z,14Z)], LysoPC [20:1(11Z)], LysoPC [16:1(9Z)/0:0)], six metabolites annotated digestive system (Glycocholic acid, L-tryptophan, Acetylcholine, Cholic acid, Prostaglandin F2a, P-cresol), four metabolites annotated signal transduction, four metabolites annotated amino acid metabolism [Phenylacetylglycine, 5-Methoxyindoleacetate, L-tryptophan, (S)-3-Hydroxyisobutyric acid], three metabolites annotated signaling molecules and interaction (S1P, Acetylcholine, Prostaglandin F2a), three metabolites annotated endocrine system (Aldosterone, Acetylcholine, Prostaglandin F2a), three metabolites annotated nervous system (including L-tryptophan, Acetylcholine, Prostaglandin F2a) (**Figure 6A**). Pathway annotation analysis by KEGG revealed the pathways where the p-value is in the top 20 (bile secretion: OS, Phospholipase D signaling pathway: EIP, sphingolipid signaling pathway: EIP, sphingolipid metabolism: M, Serotonergic synapse: OS, Neuroactive ligand–receptor interaction: EIP, primary bile acid biosynthesis: M, protein digestion and absorption: OS, Glycerophospholipid (GP) metabolism: M, regulation of actin cytoskeleton: CP, nicotine addiction: HD, Aldosterone-regulated sodium reabsorption: OS, Fc gamma R-mediated phagocytosis: OS, tuberculosis: HD, Apelin signaling pathway: EIP, African trypanosomiasis: HD, calcium signaling pathway: EIP, tryptophan metabolism: M, choline metabolism in cancer: HD, cholinergic synapse: OS (**Figure 6B**) Pathway topology analysis highlighted the following pathways: sphingolipid (SP) metabolism, tryptophan (Trp) metabolism, Glycerophospholipid (GP) metabolism, primary bile acid biosynthesis, folate biosynthesis, Aminoacyl-tRNA biosynthesis, Valine, leucine, and isoleucine degradation, phenylalanine metabolism, glycine, serine and threonine metabolism, Butanoate metabolism, arachidonic acid metabolism, Steroid hormone biosynthesis, phenylalanine, tyrosine, and Trp biosynthesis, *etc.* (**Figure 6C** and **Table 3**) Besides, SP metabolism, Trp metabolism and GP metabolism were the most important pathways according to the P-value corrected (**Table 3**).

**Table 3:** Pathway topological characteristics of 94 differentially expressed metabolites.

Pathway_ID	Pathway Description	Match_status	Num	Impact_value	P value_uncorrected	P value_corrected
map00600	Sphingolipid metabolism	2|21	2	0.024390244	0.002186261	0.0142107
map00380	Tryptophan metabolism	2|54	2	0.164478114	0.013490228	0.035074593
map00564	Glycerophospholipid metabolism	2|48	6	0.009100322	0.010828049	0.035191159
map00120	Primary bile acid biosynthesis	2|46	2	0.008546673	0.009995385	0.043313334
map00790	Folate biosynthesis	1|56	1	0	0.169623492	0.169623492
map00970	Aminoacyl-tRNA biosynthesis	1|52	1	0	0.159718126	0.17302797
map00280	Valine, leucine, and isoleucine degradation	1|40	1	0.028084852	0.128096085	0.185027678
map00360	Phenylalanine metabolism	1|51	1	0	0.157193065	0.185773623
map00260	Glycine, serine, and threonine metabolism	1|47	1	0	0.146894973	0.190963465
map00650	Butanoate metabolism	1|39	1	0.066598709	0.125328146	0.203658237
map00590	Arachidonic acid metabolism	1|37	1	0	0.119729446	0.222354685
map00140	Steroid hormone biosynthesis	1|89	1	0.0096509	0.240179458	0.223023783
map00400	Phenylalanine, tyrosine, and tryptophan biosynthesis	1|34	1	0	0.111172765	0.240874324

Pathway_ID: represents the KEGG Pathway number; Match_status: represents the metabolites participating in the pathway. The data before the “/” represents the number of metabolites participating in the pathway in the current metabolism concentration; the number behind the “/” is the total number of metabolites in the current pathway; Pathway description: represents the name of the path; IMPACT VALUE: represents the overall importance score of the pathway, with a total score of 1, which can be calculated, according to the relative position of metabolites in the pathway.

## Discussion

In the present study, a non-targeted metabolomics approach based on LC-MS was performed to explore the characteristics of blood metabolism in rats with DMMCI. Diabetes was induced by intraperitoneal administration of streptozotocin (STZ, 35 mg/kg) after 6 weeks of HFD feeding. The NORT and MWM tests were used to evaluate cognitive deficits in rats at 4 weeks or 8 weeks after DM rat model establishment. Compared to the NC and HFD 8w groups, both NOR and MWM tests indicated significant cognitive dysfunction in the DMMCI group, which could be used as an animal model of DMMCI. In metabolic profiling analysis, we identified 94 differentially expressed (44 up-regulated and 50 down-regulated) endogenous metabolites. The 10 top up-regulated and 10 top down-regulated potential biomarkers were screened according to the FDR of significance. These biomarkers by pathway topology analysis were primarily involved in the metabolism of GP metabolism, Linoleic acid metabolism, arachidonic acid metabolism, Trp metabolism, primary bile acid biosynthesis, alpha-Linolenic acid metabolism, Glycosylphosphatidylinositol (GPI)-anchor biosynthesis, SP metabolism, Folate biosynthesis, Valine, leucine and isoleucine degradation, Aminoacyl-tRNA biosynthesis, Steroid hormone biosynthesis. Therefore, our results revealed that DM could cause cognitive impairment by affecting a variety of metabolic pathways especially lipid metabolism. Besides, GP metabolism and Trp metabolism showed marked perturbations over DMMCI and could contribute to the development of disease.

DCD with cognitive impairment as the main clinical manifestation, such as learning and memory deficit, and even dementia, is a common complication of DM (1, 14). Our study observed significant cognitive decline accompanied by hyperglycemia and weight loss in HFD-fed and STZ-treated diabetic rats at 8 weeks in animal models, which is in agreement with those of previous studies (15, 16). Therefore, T2DM-8w rats were selected as the DMMCI rat model for further serum metabolomics analysis. However, it is interesting that different experiments reported different times of cognitive impairment in rats or mice (4 to 12 weeks or more). It may be due to different experimental designs, such as T1DM or T2DM, and different study specimens, such as cerebrospinal fluid, hippocampus, and urine (15–19). This animal model has also been established to explore its potential metabolic mechanisms based on the metabonomic approach between STZ-induced diabetic rats with cognitive impairment (DMMCI) and age-matched groups (NC) when they focused on changes in cerebrospinal fluid, brain tissue, or urine metabolites (19–21), but to our knowledge serum metabolomics has been rarely reported.

According to our results, T2DM induced cognitive dysfunction and significant lipid perturbations in the blood, especially in GP, SP, and Trp metabolisms which may be integral to the evolution of DMMCI neuropathology.

GPs are crucial structural components of neural membranes (predominantly including GPs, SPs, and cholesterol), which not only constitute the backbone but also maintain the membrane with a fluidity, suitable environment and ion permeability (22). The five prominent classes of GPs include phosphatidylethanolamine (PE), phosphatidylcholine (PC), phosphatidylserine (PS), phosphatidylinositol (PI) and phosphatidic acid (PA) (23). Plasma lipidomics studies in humans have also revealed a significant association between PE (consequently, a decreased PC : PE ratio) and obesity (24), prediabetes, and type 2 diabetes (25). This rearrangement can radically alter membrane potential and permeability to proteins such as cytokines. Maintaining this balance seems to have an important impact on health. Besides, they also act as a storage depot for lipid mediators derived from GPs which have been suggested to be involved in abnormal signal transduction processes, oxidative stress, neuroinflammation and neurodegeneration of AD (22). Similar to previous studies (26, 27), our results found that the levels of PE (increased), PC (increased), and their metabolites LysoPC, LysoPE (decreased) were significantly disturbed in serum compared with the control groups, suggesting that they may participate in the pathological process of cognitive impairment in diabetic rats.

Compared with GPs, SPs (such as sphingomyelins, gangliosides and ceramides) which constitute membrane microdomain “lipid rafts”, appear very low in abundance, usually being present in the body less than 20% of the level of their glycerolipid (28, 29). These lipids belong to a family of lipid molecules, circulate in the serum and accumulate in the skeletal muscle and associate with insulin resistance and glucose homeostasis. Ceramides and related sphingolipids, as mediators of insulin resistance, cell death, and inflammation (30), can interfere with insulin signaling (31), suggesting that they play an important role in DMMCI. The previous study has used quantitative and targeted metabolomics to identify a group of SPs, demonstrating that their concentrations in brain tissue correlate with neuropathological severity of AD, and in blood with measurements of pre-clinical and pro-clinical AD progression (32). In addition, more and more evidence shows that the metabolism of GPs, SPs, and cholesterol are closely interconnected and interrelated. For example, GP-derived lipid mediators (arachidonic acid) regulate SP metabolism by regulating sphingolipase, and SP-derived lipid mediators (ceramide, ceramide-1-phosphate) modulate GP metabolism by regulating the isomer of phospholipase A2 (PLA2) (33). The interaction between their metabolites may act an important role in the initiation and maintenance of oxidative stress related to neurological diseases (such as stroke, AD, and Parkinson’s disease) as well as in the proliferation, differentiation, and apoptosis of nerve cells (34). Some recently discovered SP mediators contain S1P as shown in our results (up-regulated markers) and ceramine-1-phosphate, which are key mediators of cellular reactions. S1P is a strong signaling molecule that, in addition to regulating essential physiological processes such as blood vessels, bone formation (35, 36) and inflammatory response (37), also regulates many molecular events critical to brain development and neuronal survival (38, 39). In cells, S1P may play different roles according to its subcellular localization, normally regulating mitochondrial function (40), gene expression (41), and endoplasmic reticulum (ER) stress (42); extracellularly, S1P has been shown to influence cell proliferation and migration, cell differentiation and survival, and neurite growth and neurogenesis by regulating five known G-protein-coupled receptors, S1PR1–S1PR5 (43, 44). It is hypothesized that regulation of SP metabolism and its associated signaling pathways may be a potential treatment for these devastating diseases.

Trp is a significant biosynthetic precursor of neurotransmitters, which is closely related to attention, memory, and reaction ability as a monoamine neurotransmitter (45). Trp metabolic routes consist of the two branches of the serotonin (5HT) and kynurenine pathway (KP). Trp could be metabolized into 5-HT, which promotes the formation and maintenance of synapses and affects cerebral cortex maturation. On the other hand, Trp can be metabolized to 3-HK and QUIN through the KP route, which has toxic effects on the nervous system. 3-HK could accelerate the generation of free radicals and mediates the death of neurons (21).

It was found that the content of neuroprotective 5-HT in the striatum and cortex of the aged rats decreased (46), while the contents of neurotoxic 3-HK in serum and hippocampal pyramidal neurons increased in AD patients (47). In this study, we found a similar change in the levels (decreased) of Trp in the blood of the DMMCI rats. Therefore, there is no doubt that the development of an effective means to explore the dynamics of Trp metabolic pathways in the central and peripheral systems may benefit the discovery of biomarkers for clinical treatment and pathological features of cognitive dysfunction.

Similar serum analysis to establish the link between DM and MCI has also been reported in some patient studies (48, 49). Zhang et al.’s study in 2015 on the plasma metabolomic *Profiling of Patients* has also found the disorders of sphingolipid metabolism and bile acid metabolism both happened in T2DM and diabetes-associated cognitive decline (DACD) (48). Morris et al.’s study in 2018 observed lower abundances of Trp, phosphatidylcholines (PCs), and sphingomyelins in cognitive healthy subjects with T2DM compared with those without T2DM and suggested that AD may obscure the typical metabolic phenotype of T2DM (49). These prior studies indicated that there was certain similarity/link in identified pathways/metabolites between our developed rat model and patients, which further validated the developed DMMCI rat model.

However, our study still has some limitations. First, a major “limitation” of untargeted metabolic phenotyping, which is also a major strength, is that as an unbiased metabolomic analysis, it would identify a wide range of metabolites and pathways. However, non-targeted LC-MS approaches have been proved useful and effective in the biomarker discovery stages of numerous metabolic phenotypic studies (48, 49). Differential expression of or modifications to these metabolites can provide a more reliable source of potential diagnostic biomarkers for DMMCI, which was also our main purpose. Once potential biomarkers are identified from the findings of either untargeted metabotyping studies, these can be confirmed through targeted approaches, using specific, fully validated, quantitative methods and this will be our next study direction. Second, the relatively small number of serum samples in our primary non-target metabolomics analysis may have limited our ability to probe substantial associations with other metabolites. Future studies will need more serum samples to validate and confirm our findings. Third, it should be noted that our main analysis was only based on serum metabolites, and these metabolites represent only a small part of the organism metabolome. Future analyses will expand our study framework to contain more classes of metabolites.

## Conclusion

Our study indicated that alterations in serum metabolites of lipid metabolism such as up-regulation of PE and S1P and down-regulation of PC and L-Trp may contribute to the underlying mechanisms of DMMCI by affecting GP metabolism, Trp metabolism, and SP metabolism pathways, respectively. Serum PE, PC, L-Trp, and S1P may be used as the most critical biomarkers for early diagnosis of DMMCI. An LC-MS-based metabolomics technology has potential value in identifying DMMCI biomarkers for the early detection and provides a novel avenue for effective therapeutic intervention in DCD.

## Data Availability Statement

The raw data supporting the conclusions of this article will be made available by the authors without undue reservation.

## Ethics Statement

The animal study was reviewed and approved by the Animal Ethics Committee of Central South University Xiangya School of Medicine.

## Author Contributions

YS determined the structure of the review. RC and YZ selected the references and contributed to the writing. WX and LZ contributed to the revision and finalization of the article. All authors contributed to the article and approved the submitted version.

## Funding

This study was supported by the National Natural Science Foundation of China (No. 81601141; No. 81641039), the Science and Technology Department Funds of Hunan Province Key Project (No. 2018JJ3822; No. 2019JJ50884), and the National Science & Technology Fundamental Resources Investigation Program of China (2018FY100900).

## Conflict of Interest

The authors declare that the research was conducted in the absence of any commercial or financial relationships that could be construed as a potential conflict of interest.
