# Supplementary figures and images for: LC-MS-Based Untargeted Metabolomics Reveals Early Biomarkers in STZ-Induced Diabetic Rats With Cognitive Impairment
Source: Front Endocrinol (Lausanne). 2021 Jun 30;12:665309. doi: 10.3389/fendo.2021.665309 (PMC8278747; doi:10.3389/fendo.2021.665309)

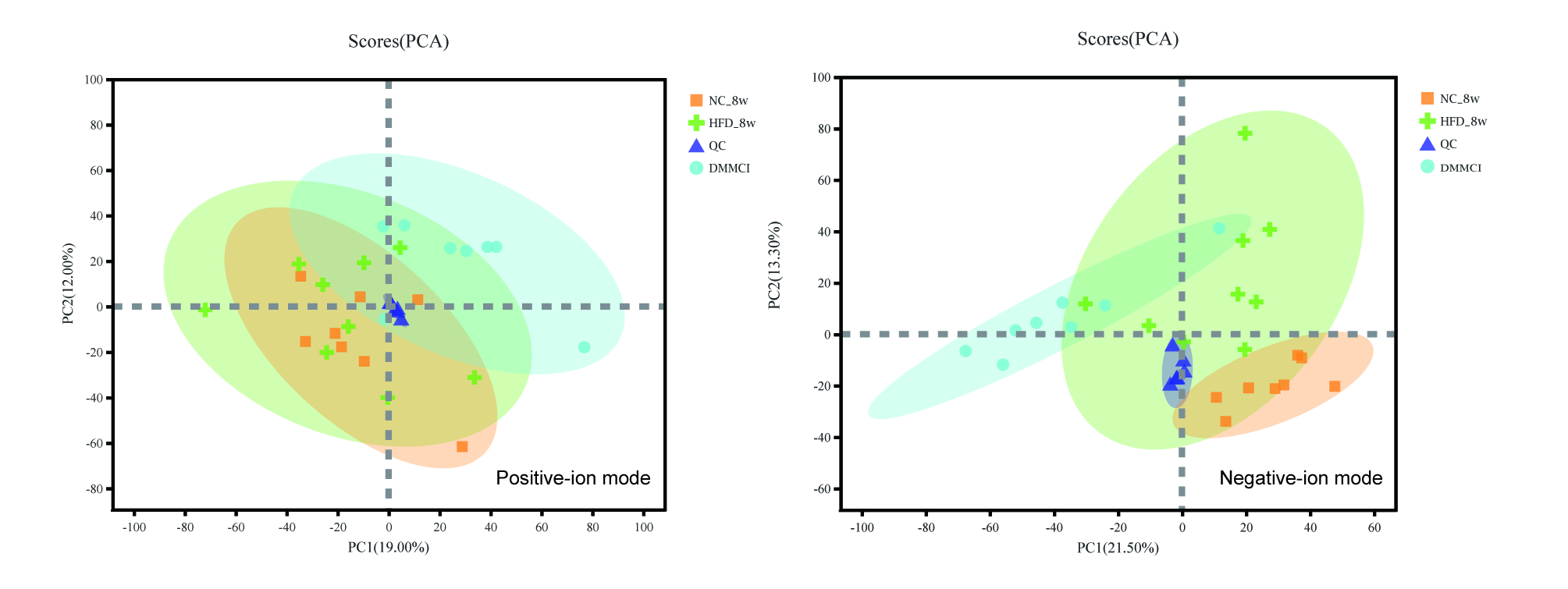


**Fig. S1** PCA score plot of QC analysis

Supplement: Supplementary file 3 [file DataSheet_3.docx]
